# B Cells and B Cell Blasts Withstand Cryopreservation While Retaining Their Functionality for Producing Antibody

**DOI:** 10.3390/cells7060050

**Published:** 2018-05-31

**Authors:** Philipp Fecher, Richard Caspell, Villian Naeem, Alexey Y. Karulin, Stefanie Kuerten, Paul V. Lehmann

**Affiliations:** 1 Research & Development Department, Cellular Technology Limited, Shaker Heights, OH 44122, USA; philipp-fecher@t-online.de (P.F.); richard.caspell@immunospot.com (R.C.); villian.naeem@immunospot.com (V.N.); alexey.karulin@immunospot.com (A.Y.K.); 2 Institute of Anatomy and Cell Biology, Friedrich-Alexander University Erlangen-Nürnberg, 91054 Erlangen, Germany; stefanie.kuerten@fau.de

**Keywords:** four color B cell ELISPOT, immune monitoring, freeze-thawing PBMC, plasma cells, antibody secretion, immunoglobulins, antibodies, immunoglobulin classes and subclasses, antibody-secreting cells, IgA, IgE, IgD, IgM, IgG1, IgG2, IgG3, IgG4, multiplex immune assay

## Abstract

In individuals who have once developed humoral immunity to an infectious/foreign antigen, the antibodies present in their body can mediate instant protection when the antigen re-enters. Such antigen-specific antibodies can be readily detected in the serum. Long term humoral immunity is, however, also critically dependent on the ability of memory B cells to engage in a secondary antibody response upon re-exposure to the antigen. Antibody molecules in the body are short lived, having a half-life of weeks, while memory B cells have a life span of decades. Therefore, the presence of serum antibodies is not always a reliable indicator of B cell memory and comprehensive monitoring of humoral immunity requires that both serum antibodies and memory B cells be assessed. The prevailing view is that resting memory B cells and B cell blasts in peripheral blood mononuclear cells (PBMC) cannot be cryopreserved without losing their antibody secreting function, and regulated high throughput immune monitoring of B cell immunity is therefore confined to—and largely limited by—the need to test freshly isolated PBMC. Using optimized protocols for freezing and thawing of PBMC, and four color ImmunoSpot^®^ analysis for the simultaneous detection of all immunoglobulin classes/subclasses we show here that both resting memory B cells and B cell blasts retain their ability to secrete antibody after thawing, and thus demonstrate the feasibility of B cell immune monitoring using cryopreserved PBMC.

## 1. Introduction

Humoral immune responses represent one of the strongest known correlates of protection against various microbial and viral pathogens, as well as toxins [1]. Traditionally, the presence of antibodies in serum has been measured for the assessment of humoral immunity. Serum antibodies, however, provide only indirect and incomplete insights into the functions of the B cell system [2]. In vivo, antibody molecules have a rather short half-life in serum: for IgG1, IgG2, and IgG4 it is 20 to 29 days, while for IgG3 it is 7 to 15 days [3]. Therefore, the presence of serum antibodies in vivo depends on their continuous production by plasma cells—immune memory that has been imprinted in the past through infection or immunization will reveal itself in serum antibody measurement only if such plasma B cells are present and continue to produce antibodies long after the antigen has been cleared [2,4].

The presence of antibodies in serum of individuals may or may not accurately reflect on the existence of humoral (or cellular) long term immunological memory (i.e., the presence of memory B or T cells in a host). On one hand, life-long antibody persistence has been documented following smallpox and flu immunizations, long after the clearance of the respective viruses [5,6]. On the other, antibody titers elicited following vaccinations against measles, tetanus toxoid, diphtheria, and poliomyelitis are known to wane over time, requiring regular booster immunizations to sustain protective antibody levels [7,8]. In yet a third scenario, memory B cells can be present in a host, capable of engaging in secondary antibody responses, however, in the absence of serum antibodies [9,10]. Thus, in addition to monitoring serum antibody levels, ascertaining the magnitude and diversity of long-lived B-cell memory populations can provide a more complete understanding of immune protection by antibodies following the re-exposure to the antigen.

The detection and enumeration of antigen-specific B cell memory cells in blood is best performed by ELISPOT [11,12]. Using this approach, not only the frequency of these cells can be established within peripheral blood mononuclear cells (PBMC) revealing the extent of their clonal sizes, but the assay is also suited to reveal the antibody classes and subclasses that these B cells produce, providing insights into the effector functions of B cell memory. While tetramers and other multimers can be used for the detection and study of rare antigen-specific T cells in PBMC [13], ELISPOT has been the primary approach for B cell immune monitoring and has been used to assess B cell memory in various antigenic and pathogenic systems [14,15,16,17]. 

During an immune response, naïve B cells, which occur in undetectably low numbers in PBMC, proliferate and differentiate into antibody secreting (B) cells (ASC), also called plasma cells, or B cell blasts. The first generation of antibodies produced by such ASC are IgM antibodies. Subsequently, immunoglobulin (Ig) class switching occurs, giving rise to ASC and memory cells capable of producing other Ig classes and subclasses. ASC in freshly isolated blood that are actively secreting antibody can only be observed during an ongoing immune encounter, and in the first weeks following the clearance of the antigen [5]. Therefore, detecting such ASC in freshly isolated blood, directly ex vivo, provides an important immunodiagnostic marker for identifying ongoing immune processes in the body vs. serum antibodies or memory B cells that in most cases do not permit to distinguish between long term immune memory and an actively ongoing antigen encounter [18]. Active B cell blasts (ASC) can be detected in the so called Direct B Cell ELISPOT assay, in which freshly isolated PBMC are plated in the assay, without additional activation. One central question that the experiments reported here addresses is whether such spontaneously secreting B cell blasts can be cryopreserved in PBMC, and subsequently thawed and cultured without disrupting their ongoing Ig secretion process. 

Upon successful elimination of an antigen, circulating ASC are no longer detectable in peripheral blood [5]. Instead, the memory B cells become resting lymphocytes that do not secrete antibody unless re-stimulated [12]. Thus, the detection of such quiescent memory B cells in PBMC requires that they be first activated to become ASC, and thus can be detected in the so called Indirect B Cell ELISPOT assay. Over the years, a variety of approaches for polyclonal B cell stimulation has been developed [19], of which a protocol reported by Pinna et al. has proven to be the most effective [19]: it relies on R848 (a TLR 7/8 agonist) and Interleukin 2 (IL-2) to achieve stimulation [20]. Comparing different protocols, we too found the combination of R848 plus IL-2 to provide the most potent polyclonal B cell stimulation (R.C., unpublished data), and used it for the present study. The second principal question addressed here is whether resting memory B cells in PBMC can be cryopreserved and subsequently thawed without disrupting their ability to re-engage in antibody production after polyclonal B cell stimulation in vitro.

B cells secrete antibodies that fall within different Ig classes, namely, IgM, IgG, IgA, and IgE, of which IgG is the most abundant in human serum. While these classes show high levels of conservation in the primary amino acid sequence, they carry key differences in their discordant Fc-regions, which contribute to their ability to bind antigen polyvalently, different tissue distributions, half-life, and overall effector functions. Functionally, IgM is associated with the initial response of naïve B cells to antigen, the IgA class protects mucosal surfaces and IgE triggers degranulation of mast cells. The bulk of the adaptive immune response to pathogens, however, is mediated by IgG which in humans occur in four subclasses: IgG1; IgG2; IgG3; and IgG4. Because each of these Ig classes and subclasses convey different effector functions it is important for immune monitoring purposes to accurately define the numbers of B cells programmed to secrete exclusively each of them (individual B cells can produce Ig molecules belonging to one class or subclass only). The original B cell ELISPOT assays were done in single color, permitting only the detection of one antibody class or subclass per test. For comprehensive immune monitoring, therefore, seven single color B cell ELISPOT assays would need to be run in parallel (if pan IgG is not measured as such, assessing the four IgG subclasses provides that information). We describe here a four color B cell ELISPOT assay that operates with the same sensitivity as single color assays, yet require one fourth of the cells, reagents, and labor. After validating this four color B cell ImmunoSpot^®^ assay, we set out to apply it to test whether PBMC can be cryopreserved before enumerating active ASC, and those reactivated by polyclonal stimulation.

Conducting immune monitoring trials based on antibody measurements in serum is rather straightforward. After the blood has been drawn, antibody molecules are stable in serum for years, and thus shipment from clinical sites to central testing laboratories does not require complex logistics, nor does the storage of the samples require sophisticated facilities and protocols to permit batch testing. In this way, testing can be done cost and labor efficiently, involving assay runs with large numbers of samples. Also, due to the stability of antibodies, the serum samples can be retested an indefinite number of times thereby facilitating not only assay optimization, development, qualification, and validation, but also the re-testing of clinical samples that have provided ambiguous results, or sharing of samples between different test facilities. In contrast, testing live PBMC for immune monitoring purposes require that the cells are either tested upon their isolation from blood, or cryopreserved in a way that they maintain their functionality upon thawing. Without the ability to cryopreserve samples effectively, clinical trials involving PBMC are close to impossible to conduct.

For decades, it was thought that PBMC could not be cryopreserved without substantial loss in function. In the meantime, protocols have been introduced that—at least for T cells—are suited to maintain the (T) cells’ functionality [21]. Loss-free cryopreservation of PBMC has permitted the development of reference sample strategies [22], and enabled the test sample logistics for conducting large scale clinical trials under GLP requirements [23]. The key to loss-free cryopreservation of PBMC lays in the understanding that it is not primarily DMSO’s toxicity, but its osmotic activity that harms the cells during the cryopreservation and thawing process [21]. According to the original protocols, ice cold DMSO is added to cells that are chilled on ice. In the chilled state, cells are metabolically inactive, however, and thus cannot actively compensate for the osmotic pressure caused by the addition of DMSO, resulting in lysis or damage to the cells. Similarly, when the cells are thawed, the original protocol requires that ice chilled washing medium is rapidly added to the thawing cells when the “last ice crystal” is still visible, that is, at a time when the cells are still chilled and metabolically inactive. Under these conditions again, the cells cannot actively compensate for the rapid change in osmotic pressure as the concentration of DMSO drops from 10% to essentially zero in seconds as the ice cold washing medium is added rapidly—again, the cells lyse or become damaged. In the new protocol, warm DMSO is slowly added to warm PBMC during the cryopreservation protocol, and when thawing, the cells are brought to 37 °C before warm washing medium is added slowly, permitting the cells to actively compensate for the change in osmolarity as the concentration of DMSO gradually changes. Using the latter protocol, measuring T cell functionality, no difference can be seen when testing fresh versus cryopreserved PBMC [21]. While this notion is widely accepted and utilized in the meantime for T cell measurements, it is still controversial whether B cells too can be cryopreserved without loss of function. We have addressed here this question for B cells, utilizing the protocol that has proven successful for T cells.

When cryopreserving B cells, one needs to distinguish between two fundamentally different activation states of these cells. Memory B cells are resting lymphocytes (with a rudimentary cytoplasm) that operate at minimal metabolic activity: as described above, such resting memory B cells can be detected only in Indirect B Cell ELISPOT Assays after they have been activated in vitro through a four day polyclonal stimulation culture to become B cell blasts that secrete antibody. Successful cryopreservation of resting memory B cells therefore requires that, after thawing, these cells can proliferate and differentiate to become ASC.

B cell blasts (ASC), in contrast, are highly activated lymphocytes that actively synthesize and secrete antibody molecules (and because of their high protein-synthesis activity they have an enlarged cytoplasm with a highly developed ER, which inspired them being named plasma cells, and plasma blasts). As they constitutively secrete antibody without the need for additional stimulation, they can be detected in Direct B Cell ELISPOT Assays. As B cell blasts are present in ex vivo PBMC only during ongoing infections, or shortly after vaccinations, they can rarely be detected in unstimulated PBMC of healthy donors. The PBMC library we tested was from healthy donors in whom we could not detect spontaneous ASC using the Direct B Cell ELISPOT Assay approach. Therefore, to be able to test whether B cell blasts can be frozen without loss of function, we cryopreserved PBMC at the end of the four day polyclonal stimulation culture, when B cell blasts/ASC are abundant, and compared the ASC numbers before and after thawing these cells (See Figure S1). 

## 2. Materials and Methods

### 2.1. Human Subjects and PBMC

All 15 human subjects tested in this study were healthy adults ages 22–45 and were recruited as a part of larger PBMC banking involving over 298 individuals at Cellular Technology Ltd. (CTL, Cleveland, OH, USA). Blood donors were recruited by Hemacare (Van Nuys, CA, USA) under Hemacare IRB where the PBMC were isolated by leukopheresis. The PBMC were either tested without cryopreservation upon receipt of the cells as “fresh cells”, or were cryopreserved as described below and stored in vapor phase liquid nitrogen until testing in ELISPOT assays as “thawed cells”. The PBMC had been stored between 2 weeks and 13 years before testing, during which, at least as far as T cell functionality goes, no change was seen upon repeated testing of aliquots over time. Detailed methods of PBMC thawing, washing, and counting of the cryopreserved PBMC have been previously described [24]. PBMC were transferred to polyclonal B-cell stimulation cultures within 2 h of thawing for subsequent use in ELISPOT assays without involving “overnight resting”, as we did not find that to be of any advantage [25].

### 2.2. Polyclonal B-Cell Stimulation

B cells do not spontaneously secrete antibody. Therefor their detection requires polyclonal stimulation for several days, during which resting memory B cells differentiate into ASC that can be detected in B cell ELISPOT assays [20]. For polyclonal stimulation, fresh, or freshly thawed PBMC were resuspended in CTL-Test B™-media (CTLTB-010, CTL, Cleveland, OH, USA) supplemented with polyclonal B cell stimulator (CTL-BPOLY200, CTL, which contains R848 and human IL-2 and is part of the ImmunoSpot^®^ kits) according to the manufacturer’s instructions. The PBMC were at 4 million cells/mL, in 25 cm^2^ tissue culture flasks and were cultured in an incubator at 37 °C, 5% CO_2_ for four days.

### 2.3. Cryopreservtion of PBMC

For cryopreservation of PBMC osmotic pressure caused by DMSO (more than DMSO’s intrinsic toxicity) is one of the primary factors that needs to be controlled. Therefore, all reagents should be used at 37 °C. Freezing followed CTL’s protocols and using CTL’s reagents, as follows. In preparation, CTL-Cryo™ A was mixed with CTL-Cryo™ B in an 80% to 20% (*v*/*v*) ratio (4:1), by slowly adding CTL-Cryo™ B into CTL-Cryo™ A. The resulting CTL-Cryo™ A-B mixture and CTL-Cryo™ C were warmed up to 37 °C in a CO_2_ incubator. The PBMC to be cryopreserved were resuspended PBMC in warm CTL-Cryo™ C medium adjusting the cell concentration to 20 × 10^6^ cells/mL, and also placed for 20 min into the incubator at 37 °C. After these preparations, the admixing of the warmed-up cells and the warmed up freezing solution began. The cells were resuspended by gently tapping the tube and slowly, over a time period of approximately two minutes, an equal volume of warm Cryo™ A + B was added, drop-by-drop while gently whirling the tube to ensure complete mixing of the two solutions. Next, the cells were aliquoted into pre-labeled cryovials while pipetting them gently and slowly to minimize shear forces. Next, the cryovials were placed into a room temperature Nalgene^®^ cryofreezing container (Mr. Frosty, Thermo Scientific, Watham, MA, USA) filled with propanol and transferred into a −80 °C freezer for 24 h, after which the vials were transferred into vapor/liquid nitrogen tanks for long term storage.

The thawing of PBMC also followed CTL protocols using CTL reagents. On the day of the experiment, the cryovials were pulled from the liquid nitrogen tanks, and transported on dry ice to the laboratory, where they were immediately placed into a 37 °C water bath for 10 min. During this time, the cells reached 37 °C temperature. Next, the cells were aspirated from the cryovial, pipetting slowly and avoiding shear forces, and transferred into a 50 mL conical tube. To recover the residual cells from the cryovial, 1 mL of 37 °C warm CTL Anti-Aggregate Wash™ Medium was added to each cryovial, aspirated, and added slowly to the rest of the cells. This resulted in the first 1 + 1 dilution of DMSO. Slowly, additional 37 °C warm CTL Anti-Aggregate Wash™ Medium was added to the 50 mL tube while gently swirling the tube: the first 3 mL were added over one minute, followed by 1 mL over five seconds until the cells were resuspended in 10 mL, and thus DMSO has been diluted 1 in 10. Additional 20 mL of warm CTL Anti- Aggregate Wash™ Medium was added over 1 min. The cells were now spun in at 330 g for 10 min at room temperature, with rapid acceleration and the brake on. After decant the supernatant, the pellet was carefully resuspended by tapping the tube (avoid pipetting or vortexing). Ten ml of 37 °C CTL Anti-Aggregate Wash™ Medium was added, then the cells were mixed by inverting the tube twice 180° with the cap tightly closed. A cell sample was taken for cell counting by the CTL’s Live/Dead/Apoptotic cell counting suite using a CTL S6 Ultimate Analyzer (by CTL, Cleveland, OH, USA). After one more washing step using warm CTL Anti-Aggregate Wash Medium, the cells were adjusted to the desired cell concentration in warm CTL-Test™ Medium, and stored in the CO_2_ incubator until plated into the assays.

### 2.4. B-Cell ELISPOT Assays

After four days of polyclonal stimulation, the cells were counted with CTL’s Live/Dead/Apoptotic cell counting suite using a CTL S6 Ultimate Analyzer. After washing the cells one time, the cells were adjusted to 2.5 × 10^6^ cells/mL, of which the specified number of cells was plated, in serial dilutions and in quadruplicates, into 96-well plates that were pre-coated with anti-κ/λ capture antibody contained in the kit. Human Four-Color ImmunoSpot^®^ IgM/IgG/IgA/IgE, Three-Color Human ImmunoSpot^®^ IgM/IgG/IgA, and Human Four-Color ImmunoSpot^®^ IgG1/IgG2/IgG3/IgG4 assay kits were used, as specified, all from CTL. The cells were incubated for a period of 24 h at 37 °C, 5% CO_2_ during which the antibodies that the B cells secreted were captured on the membrane by the capture antibody directly around the secreting cells. Thereafter, the plates were decanted and washed three times with 200 µL sterile PBS. The plate-bound Ig “spots”, each representing the secretory foot print of a single ASC, were visualized using the four anti-Ig class/subclass-specific detection antibodies contained in the kits, following the manufacturer’s specifications.

### 2.5. Plate Reading and Statistical Analysis

Following the completion of the B cell ELISPOT assay, the plates were air-dried in a laminar flow hood prior to being scanned and counted on an ImmunoSpot^®^ S6 Ultimate Reader. The numbers of B cell Spot Forming Units (SFU), each corresponding to the secretory foot print of an ASC, were established using the BasicCount™-Mode of the ImmunoSpot^®^ Software. Means and standard deviations were calculated for the quadruplicate wells, tested for each test condition. For each PBMC donor, class and subclass, and for fresh, thawed and blast cells of each, the linearity of spot counts was established, based on which the SFU counts were extrapolated to SFU/million using standard functions of Excel. The Student’s *t*-test, or the ANOVA test were performed to detect statistical differences between two or three groups of Spot Forming Unit (SFU) counts, respectively.

## 3. Results and Discussion

### 3.1. Developing Four Color B Cell ImmunoSpot^®^ Assays

**Figure 1:**
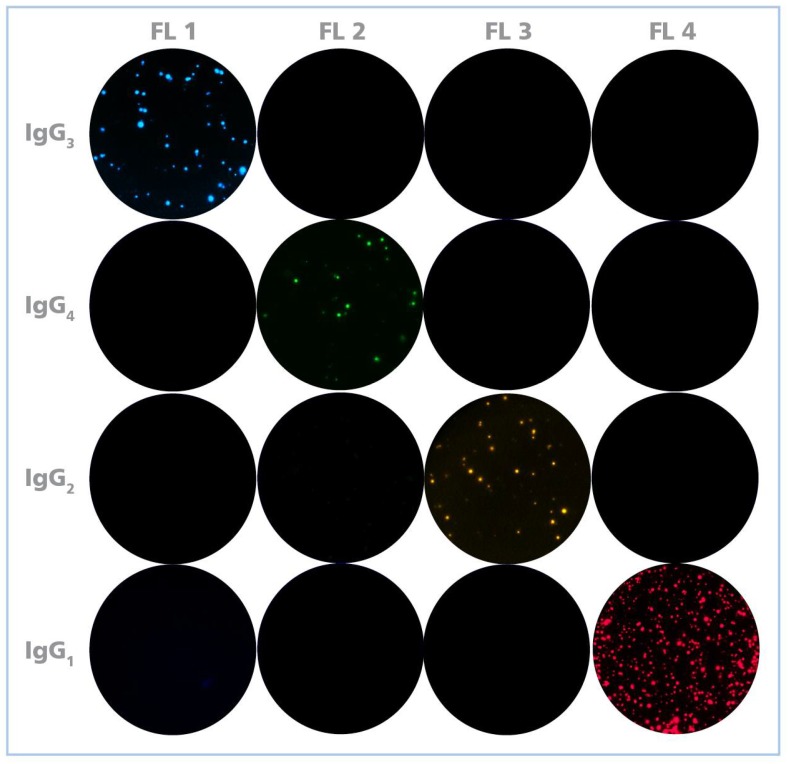
Unambiguous detection of the four fluorescent tags identifying the individual IgG subclasses. In each row, a single-color B cell ELISPOT assay was performed using the respective IgG subclass-specific detection reagent, as specified in the figure. Each assay was analyzed with filter settings optimized for the individual colors. Across three independent experiments (of which representative scans are shown), individual florescent channels (FL1 to FL4), readily detected corresponding fluorescent tags. Importantly, no cross-bleeding of colors between channels occurred.

**Figure 2:**
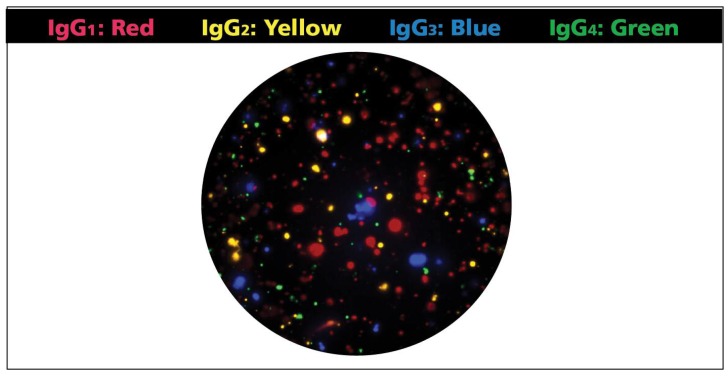
Representative four-color well. The membrane was coated with anti-κ/λ capture antibody and polyclonally activated peripheral blood mononuclear cells (PBMC) were plated at 50,000 cells/well. Detection regents for each of the four IgG subclasses were added to visualize the membrane-bound IgG molecules. Images were captured for each individual fluorescent channel (see Figure 1) and superimposed using the following artificially assigned colors for each color plane: IgG1: Red; IgG2: Yellow; IgG3: Blue; and IgG4: Green.

One of the primary challenges when developing fluorescence-based multicolor assays is avoiding that the individual fluorochromes’ signal cross bleeds between fluorescent channels (filters) resulting in false positive signals that needs to be corrected—if possible at all—by compensation (like in flow cytometry). As the spectra of common organic fluorochromes that could be used for four color analysis overlap (see Figure S2), we attempted to come up with a combination of fluorochrome labels that, along with distinct excitation wavelengths and emission filters for each, permit detection of the individual analytes without the cross-bleeding of colors. Figure 1 shows that this was successfully accomplished by the Four Color ImmunoSpot^®^ system. Thus, in this four color format, unambiguous identification of the four analytes is possible by serial imaging of a single well in four different fluorescent channels. Superimposition of the four images then allows to visually display the results for each well in a multi-color format (Figure 2). As B cells secrete only one type of Ig class or subclass, the color planes can be analyzed independent of each other.

### 3.2. Four Color B Cell ImmunoSpot^®^ Assays Have Similar Sensitivity for Detection of the Individual Antibody Classes/Sub-Classes as the Corresponding Single Color Assays

ELISPOT assays have been traditionally performed using precipitating enzymatic substrates. The enzymatic reaction not only serves as an amplification step, but also permits image acquisition using relatively simple reflected white light systems. We have addressed experimentally whether the sensitivity for detecting analytes using the Four Color ImmunoSpot^®^ platform would match that of traditional enzymatic assays. The same polyclonally pre-stimulated PBMC were plated into wells coated with the same anti-k/λ capture antibody pair. After a 24 h cell culture period, during which the antibodies secreted by the ASC were captured, either (a) the four fluorescence-tagged antibodies were added for simultaneous detection of all four IgG subclasses in the four color format; or (b) the individual fluorescent-tagged antibodies were added separately for single color fluorescent detection; while in parallel (c) the corresponding single color enzymatic ELISPOT assays were performed. The spots obtained under all three assay conditions were counted, and compared (Table 1). The results showed that, for each of the four analytes, the spot counts were similar, with the slight differences seen not reaching statistical significance as per the ANOVA test.

### 3.3. Intermediate Accuracy of Four Color ImmuoSpot^®^ B Cell Assays

**Figure 3:**
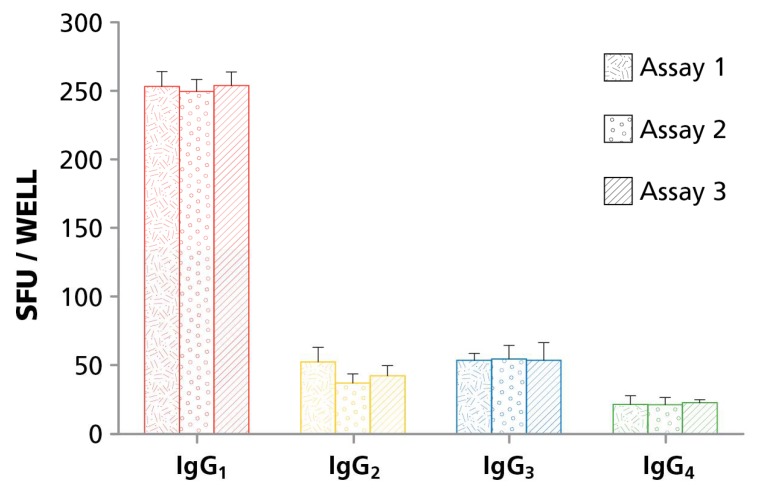
Reproducibility of the four color B cell assay. To assess inter-assay variation in spot numbers, PBMC were stimulated polyclonally, and frozen in aliquots on day four. Three independent assays were performed after thawing an aliquot, measuring the four IgG subclasses on anti-κ/λ capture antibody-coated membranes. Each bar represents the mean spot count + SD for the tests done in four replicate wells.

Intermediate Accuracy refers to the reproducibility of results when the same investigator performs the test repeatedly. Because the following experiments had been done with cryopreserved PBMC, we could apply a reference sample strategy: by thawing different aliquots of the same donor’s PBMC that had been frozen at the same time, we could test the same ASC repeatedly. PBMC donors were selected whose cells in screening experiments showed SFU counts between 20 and 300 ASC per well, at 30,000 PBMC/well, for all four IgG subclasses. These PBMC were thawed, cultured for four days with R848 and IL-2, and on the fourth day cryopreserved in 10 aliquots. On three consecutive days, one aliquot of these pre-activated PBMC was thawed, and the cells were seeded into a four color ImmunoSpot^®^ assay. The tests were done by the same investigator (R.C.). The results reproduced satisfactorily in the three experiments for IgG1, IgG2, IgG3, and IgG4 alike, without showing significant differences in SFU counts for the three tests (Figure 3). Four Color ImmunoSpot^®^ B cell assays for IgG subclass determinations have, therefore, a very high inter-assay reproducibility.

### 3.4. Detecting Resting Memory B Cells in Thawed, Cryopreserved vs. Freshly isolated PBMC

Having established that we can precisely measure the frequencies of ASC in four color format, we set out to address the question whether resting memory B cells can be cryopreserved without losing their ability to differentiate into ASC following polyclonal stimulation, and thus becoming detectable as ASC in B cell ELISPOT assays. PBMC from 15 donors were tested, both, freshly isolated, and upon thawing following cryopreservation with the results compared for each donor under both conditions. The reference value against which the cryopreserved cells were compared was the fresh PBMC, i.e., cells that were exposed to polyclonal stimulation within hours of obtaining the cells, and cultured with R848 and IL-2 for four days, then seeded into the ELISPOT assays in which the secreted antibodies were captured for 24 h, after which the ASC numbers were established for the individual Ig classes and IgG subclasses. We did not include IgE measurements in these experiments because our previous studies showed that in healthy donors IgE producing ASC can be detected only when in vitro class switching is forced culturing PBMC for 7 days in the presence of IL-4 and anti-CD40 according to protocols published by Jabara et al. [26]. Therefore a three color IgM/pan-IgG/IgA assay was done for the Ig classes, and a four color IgG1/IgG2/IgG3/IgG4 assay was done for the IgG subclasses.

**Figure 4:**
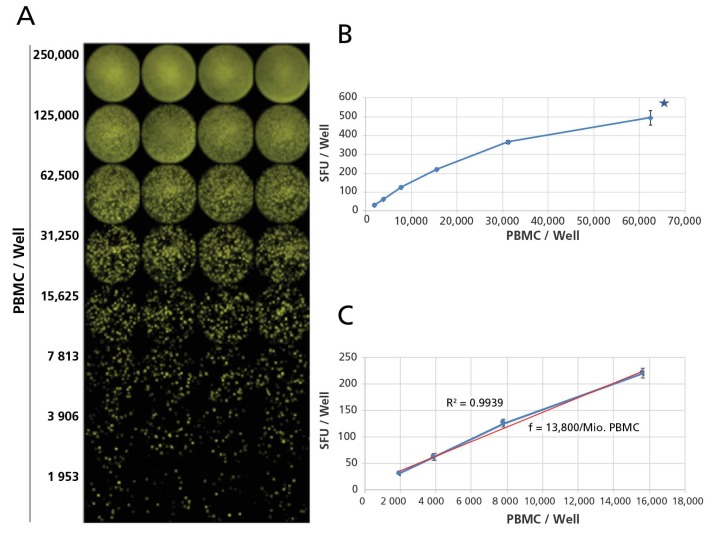
Calculating frequencies for pan IgG-SFU/million PBMC illustrated. Polyclonally stimulated PBMC were plated in the specified cell numbers in quadruplicate wells, and a Three Color ImmunoSpot^®^ assay was performed for Ig classes pan-IgG, M and A. (**A**) Raw images for the IgG color plane are shown with the cell numbers plated per well specified; (**B**) the mean spot count for each cell concentration, with the SD for the quadruplicate wells tested is plotted vs. the cell numbers plated. As the spots at high ASC densities start crowding (plus when the number of ASC is high, there is an “ELISA” effect with analyte captured from the supernatant causing a high background carpet staining), for high cell numbers Spot Forming Units (SFU) become no longer precisely countable: “too numerous to count is shown by a star; (**C**) While at high numbers spot counts tend to deviate from linearity due to crowding, in lower numbers they followed a liner relationship between cell numbers plated and SFU counted, in this case with an *R*^2^ value of 0.9939. Based on the regression line shown in red, the pan-IgG SFU frequency has been calculated to be, in the example shown here, 13,800 pan-IgG SFU per one million PBMC.

The frequencies of B cells secreting the above Ig was established within one million PBMC, by extrapolation. The PBMC were plated in one-to-two serial dilution steps, with cell numbers ranging from 2.5 × 10^5^ to 1.95 × 10^3^ PBMC per well, as frequencies of ASC for the different Ig classes and subclasses can be vastly different. Representative examples of such serial dilutions are shown for pan-IgG, IgM, IgA, and the four IgG subclasses in Figure 4 and Figures S3–S5, respectively. From such serial dilutions, wells could be chosen in which the ELISPOTs were neither overcrowded (thus no longer precisely countable), nor occurring in too low numbers. From each dilution series, four consecutive cell dilutions were chosen to (a) verify the linearity of the SFU counts vs. the cell numbers plated; and (b) to extrapolate the frequency of ASC per million PBMC as the final number for reporting results, and for the frequency comparisons.

**Figure 5:**
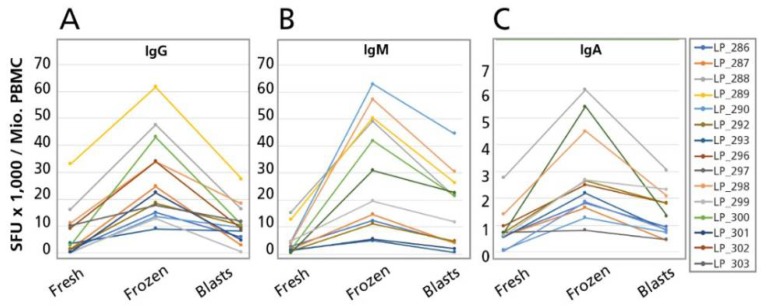
Effect of cryopreservation on Ig class production by B cells. Freshly isolated PBMC were polyclonally stimulated for four days and then tested in a three color B cell ImmunoSpot^®^ assay, with serial dilution of the cells in four replicate wells, (“Fresh”), as illustrated in Figure 4. The same PBMC were cryopreserved, thawed, and then polyclonally stimulated, seeded and tested as above (“Frozen”). The latter cells were cryopreserved at the end of the four day polyclonal stimulation culture, when B cell blasts have been engaged, then thawed, and seeded and tested as above (“Blasts”). The relationship of Fresh/Frozen and Blast cells is graphically illustrated in Figure S1. The results obtained testing 15 PBMC donors are shown here for pan-IgG (**A**), IgM (**B**), and IgA (**C**). The SFU counts per million PBMC have been established as specified in Figure 4. For each donor, as defined by color in the insert, the mean spot counts are connected by the corresponding color coded lines.

**Figure 6:**
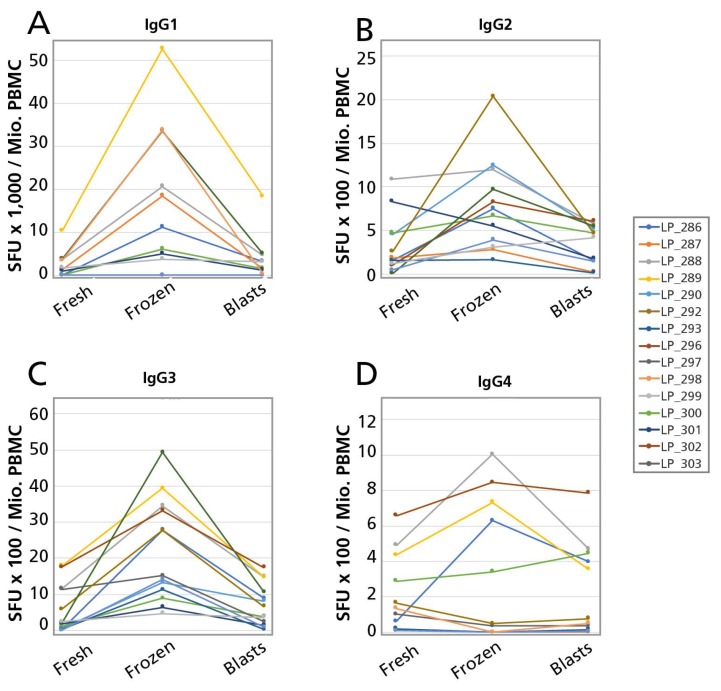
Effect of cryopreservation on IgG subclass production by B cells. The legend to Figure 5 applies except that a four color B cell ImmunoSpot^®^ assay was performed measuring IgG1 (**A**), IgG2 (**B**), IgG3 (**C**), and IgG4 (**D**) on the serially diluted PBMC. Results obtained for 15 PBMC donors tested are shown.

The results of the ASC frequency comparisons for the fresh vs. cryopreserved cells of the 15 PBMC donors tested are summarized for the Ig Classes in Figure 5, and for the Ig subclasses, in Figure 6. The frequencies are expressed as ASC per million, calculated for each sample having been established in serial dilutions, as described above. As can be seen in these figures, in all cases when ASC were detected in the fresh cells, ASC were also detected in the corresponding cryopreserved PBMC, occurring in at least the same numbers, and typically in higher numbers for the cryopreserved cells. Whereas, by statistical analysis, the difference between fresh vs. frozen SFU counts were in each case highly significant, the difference manifested itself at a constant rate of 3–5 × higher for the frozen cells. Therefore, resting memory B cells can be cryopreserved, maintaining their ability to develop into ASC upon a four day polyclonal stimulation culture (during which they tend to expand even more that freshly isolated memory B cells) and this notion holds for all memory B cell sub-lineages: those that have not undergone class switching yet and secrete IgM, as well as those that have done so, and secrete all the other Ig classes and IgG subclasses. (We could not address this question for IgE, see above.).

### 3.5. Detecting Active Ig Secreting B Cell Blasts Following Cryopreservation

The previous experiments addressed whether resting memory B cells could be cryopreserved while maintaining their ability to differentiate into antibody-producing B cell blasts upon thawing. Unlike such dormant memory B cells, B cell blasts display high metabolic activity as their protein (including Ig-) synthesis machinery is operating at a very high rate. Such B cell blasts, therefore, might be more vulnerable to freeze-thawing, as compared to resting memory B cells. To address this possibility, we cryopreserved four day polyclonally stimulated PBMC that contain active ASC (see also Figure S1). By comparing the numbers of ASC detected in these cells before and after freeze-thawing, we could establish whether they are affected by cryopreservation and/or the subsequent thawing process. Such cryopreserved cells were thawed, then transferred to a plate for the multi-color B cell ImmunoSpot^®^ assays for 24 h in medium alone, without additional stimulation. The fresh B cell blasts were tested in an identical way prior to cryopreservation and the results were compared to the cryopreserved cells. The results are shown for the Ig classes in Figure 5, and for the IgG subclasses, in Figure 6. For all 15 test subjects, and for all Ig classes and subclasses, spontaneously secreting ASC were detected in samples after thawing if they were present before cryopreservation. The hierarchies also closely matched up: if a donor had elevated spontaneous ASC numbers prior to cryopreservation relative to another donor, that relationship was proportionally maintained in the freeze-thawed samples. However, approximately half of the ASC numbers were detected in the freeze thawed samples, compared to the numbers before cryopreserving the cells. This observation was made for all Ig classes and IgG subclasses. By statistical analysis, the difference between fresh vs. frozen SFU counts were in each case highly significant, the difference manifested itself at a constant rate of approximately half for the frozen cells. It should be noted that in these experiments the B cells underwent two freeze-thaw cycles, and that most cells are thought not to survive two freeze thaw cycles. Therefore, the results might be even more encouraging with B cell blasts frozen only once. But even after two cycles, B blasts largely maintaining their active secretory activity.

## 4. Conclusions

The main goal of B cell immune monitoring is to establish: (a) whether spontaneously antibody secreting B cell blasts are present in PBMC at their isolation, detectable in Direct ELISPOT assays; (b) whether clonally expanded memory B cell populations are present in the test individual; and (c) what type of antibody classes/subclasses these memory B cells secrete upon re-stimulation. Testing polyclonally stimulated B cells, our data show that spontaneously antibody secreting B cell plasma blasts can be detected in cryopreserved PBMC, although their numbers will likely be reduced two to three-fold. In spite of this reduction in numbers, the mere presence of spontaneously secreting ASC in cryopreserved PBMC per se cells should reveal actively ongoing immune responses because active ASC are absent in blood in a quiescent immunological state. Clonally expanded antigen-specific B cell memory populations should also be readily detectable in cryopreserved PBMC because in naïve individuals no antigen-specific B cells can be detected even after polyclonal stimulation. Therefore, in spite of the increased frequencies of ASC detected in cryopreserved PBMC, detectable numbers of ASC per se identify that B memory cells existed in vivo in clonally expanded sizes. Moreover, recently primed B cells can only secrete IgM; it takes Ig class switching in the course of an immune response in vivo to generate (memory) B cells capable of secreting any antibody class other than IgM. Therefore, detecting antigen-specific B cells in PBMC that secrete different antibody classes/subclasses in ELISPOT assays suggests that these B cells have undergone class switching in vivo. Thus, these data suggest that qualitative markers of B cell immunity are maintained in cryopreserved PBMC. This notion was confirmed in a recent study of HCMV-specific T and B memory cells [27]. Testing cryopreserved PBMC of 82 healthy human donors we found that most individuals who possessed HCMV-specific CD4- and CD8 memory T cells in increased frequencies also possessed HCMV-specific B cells in increased frequencies. Notably, all these B cells secreted IgG1 antibody, the Ig subclass that prevails in sera of subjects with latent HCMV infection [28]. In contrast, in test subjects in whose cryopreserved PBMC HCMV-specific memory T cells were undetectable, B cells producing HCMV-specific antibodies were also absent, and this finding held up for all Ig classes and subclasses [27]. In this study, we tested for antigen-specific ASC, that is, by coating the membrane with the antigen itself. An elegant alternative approach for detecting antigen-specific ASC has been introduced by Jahnmatz et al. [29] in which first all antibodies secreted by ASC are captured on the membrane and the fluorescence labelled antigen is added to visualize the antigen-specific ASC. Our data predict that using either approach the moderate increase of ASC numbers in cryopreserved PBMC should be rather predictable and proportional among donors. If a donor had low SFU counts relative to another donor in the fresh cells, that relationship is maintained proportionally in the cryopreserved cells. This notion represents a major advancement for the perspective of B cell immune monitoring with cryopreserved PBMC compared to the alternative that it can be done reliably with fresh PBMC only. The use of cryopreserved PBMC enables cost-effective, large scale immune monitoring that can be adapted to regulated work, including developing reference PBMC strategies.

Presently, it is unclear why the ASC numbers increase in cryopreserved cells. One possibility is that B cells survive cryopreservation better than other cell populations in PBMC. If such a difference exists, it is not detectable right after thawing of the cells at which time points we compared the fresh vs. freeze-thawed cells (data not shown). Thus, the difference in ASC numbers manifests itself during the four days polyclonal stimulation culture. During these four days, responding to polyclonal stimulation, the resting B cells need to undergo blast transformation and to activate their Ig synthesis machinery. In addition, these B cells will engage in proliferation. All these cellular events are under the influence of a microenvironment defined by cytokines and cell surface molecules contributed of bystander cells present in the PBMC. Thus, the increased ASC numbers after cryopreservation could either result from a direct effect on the B cells, a possibility that these authors consider more unlikely, or could occur as a consequence of affecting third party cells, e.g., cells of the innate immune system that, like the B cells themselves, also become activated by the TLR agonist R848 and IL-2, and apparently responded to these stimuli differently in freeze thawed PBMC, thus providing a modified (more co-stimulatory) microenvironment for B cell proliferation and differentiation than do third party PBMC in freshly isolated PBMC. Another -yet related possibility is that regulatory cells do not withstand freeze thawing, and thus do not exert an inhibitory effect in the cryopreserved PBMC samples.

## Figures and Tables

**Table 1:** Single-color (SC) enzymatic and SC fluorescent B cell ELISOT assays performed to detect individual IgG subclasses provide the same antibody secreting (B) cells (ASC) frequencies as the corresponding four-color (4C) ImmunoSpot^®^ assay. Polyclonally stimulated B cells were seeded into SC enzymatic B cell ELISPOT assays that detected the four IgG subclasses. In parallel, SC fluorescent assays were performed for each IgG subclass, and a Four Color ImmunoSpot^®^ assay was performed. The three different assays were each performed in four replicate wells. The spot numbers for each IgG subclass were counted, with the mean and standard deviation (SD) of the replicates shown.

	IgG1	IgG2	IgG3	IgG4
SC Enzymatic	241 ± 13	60 ± 8	67 ± 8	29 ± 7
SC Fluorescent	248 ± 16	59 ± 3	43 ± 5	25 ± 3
4C Fluorescent	250 ± 9	57 ± 7	55 ± 10	21 ± 5

## Supplementary Materials

Supplementary materials are available on line http://www.mdpi.com/2073-4409/7/6/50/s1.
