# Supplementary material for: B Cells and B Cell Blasts Withstand Cryopreservation While Retaining Their Functionality for Producing Antibody
Source: Cells. 2018 May 31;7(6):50. doi: 10.3390/cells7060050 (PMC6028916; doi:10.3390/cells7060050)
Supplement: Supplementary file 1 [file cells-07-00050-s001.pdf]

B Cells and B Cell Blasts Withstand Cryopreservation While Retaining Their Functionality for Producing Antibody

Philipp Fecher[1, 2], Richard Caspell[1], Villian Naeem[1], Alexey Y. Karulin[1], Stefanie Kuerten[2] and Paul V. Lehmann [1,*]

1 Research & Development Department, Cellular Technology Limited, Shaker Heights, OH 44122, USA; philipp-fecher@t-online.de (P.F.); richard.caspell@immunospot.com (R.C.); villian.naeem@immunospot.com (V.N.); alexey.karulin@immunospot.com (A.K.); paul.lehmann@immunospot.com (P.V.L.)

2 Institute of Anatomy and Cell Biology, Friedrich-Alexander University Erlangen-Nürnberg 91054 Erlangen, Germany; stefanie.kuerten@fau.de (S.K.)

* Correspondence: paul.lehmann@immunospot.com; Tel.: +1-216-965-6311

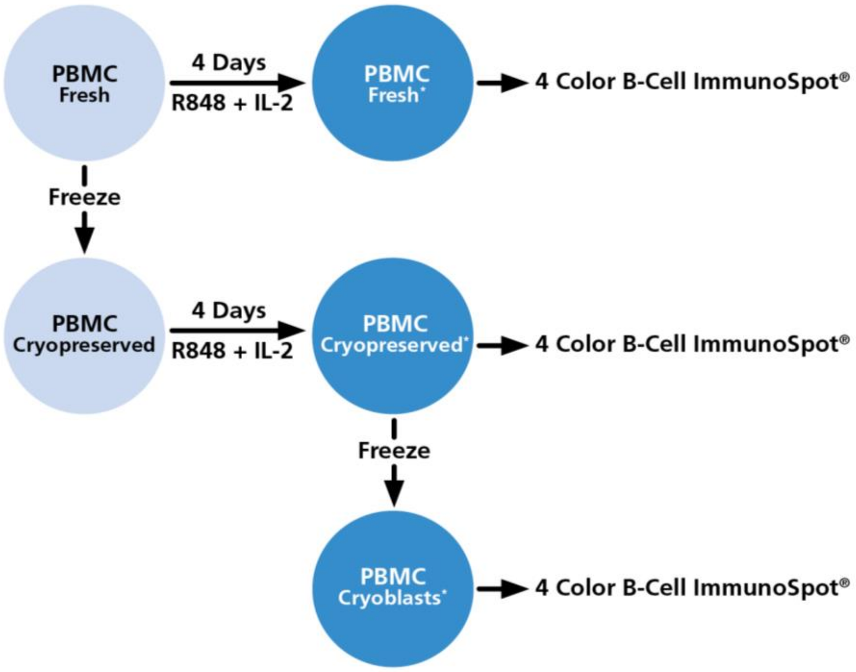

Figure S1. Comparing fresh and cryopreserved PBMC – specifying the treatment of the cells tested. Freshly isolated PBMC were polyclonally stimulated for 4 days and then seeded into a 4 color B cell ImmunoSpot® assay: "Fresh" PBMC. Upon receipt, a fraction of the fresh cells was cryopreserved, stored, thawed, and then polyclonally stimulated, now termed cryopreserved, or "Frozen" PBMC. A fraction of these "frozen" PBMC, containing B cell blasts, was re-frozen, stored, thawed and seeded without additional stimulation into ELISPOT assays as "Blasts".

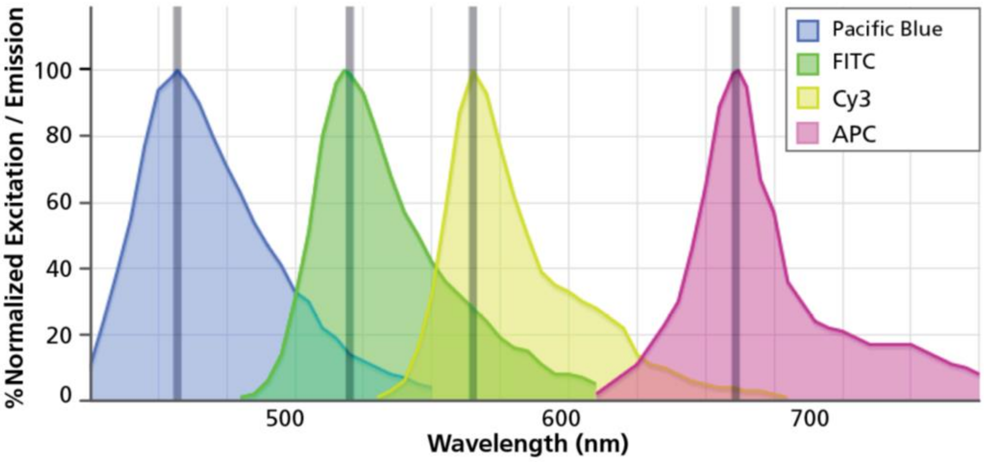

Figure S2. Partially overlapping spectra of standard organic fluorochromes. The figure shows the spectra for four standard organic fluorochromes, specified in the insert by color.

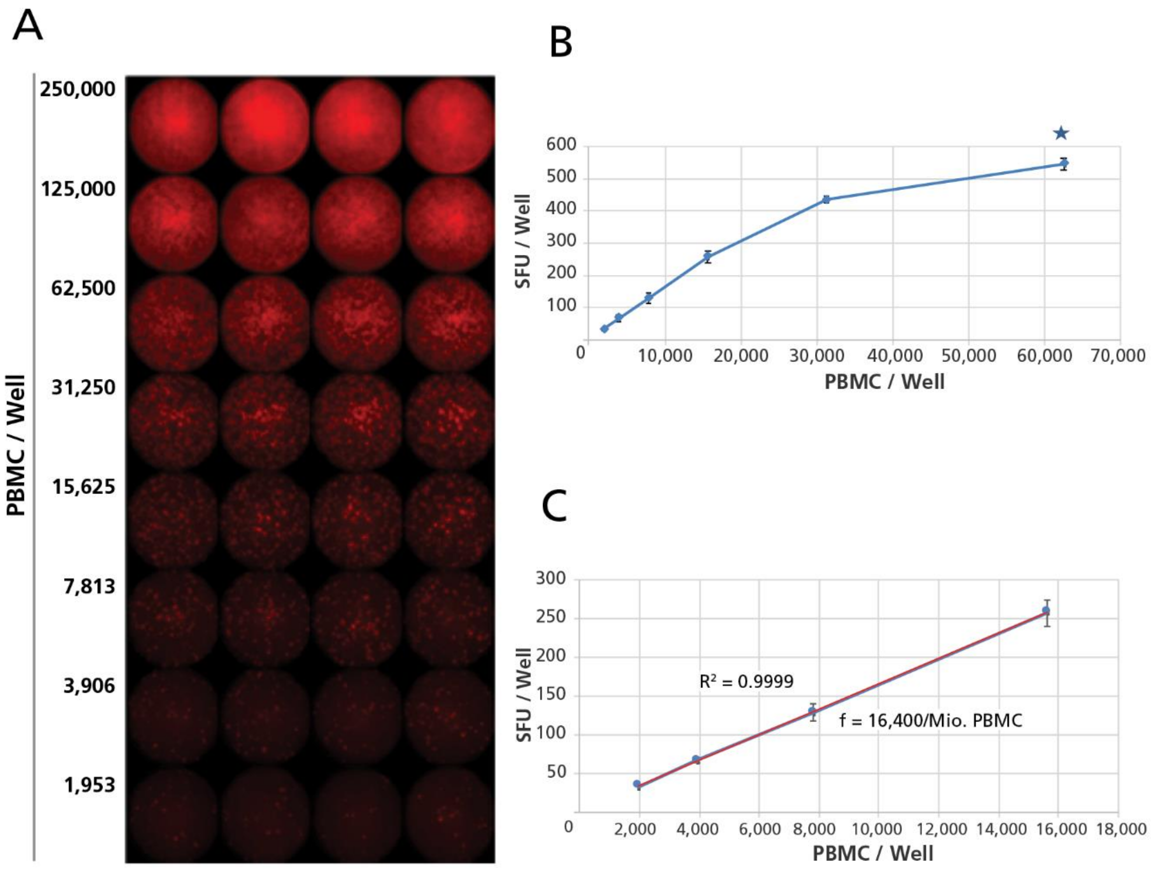

Figure S3. Calculating frequencies for IgM ASC in PBMC illustrated. The legend to Figure 4 applies, except here IgM was detected and analyzed.

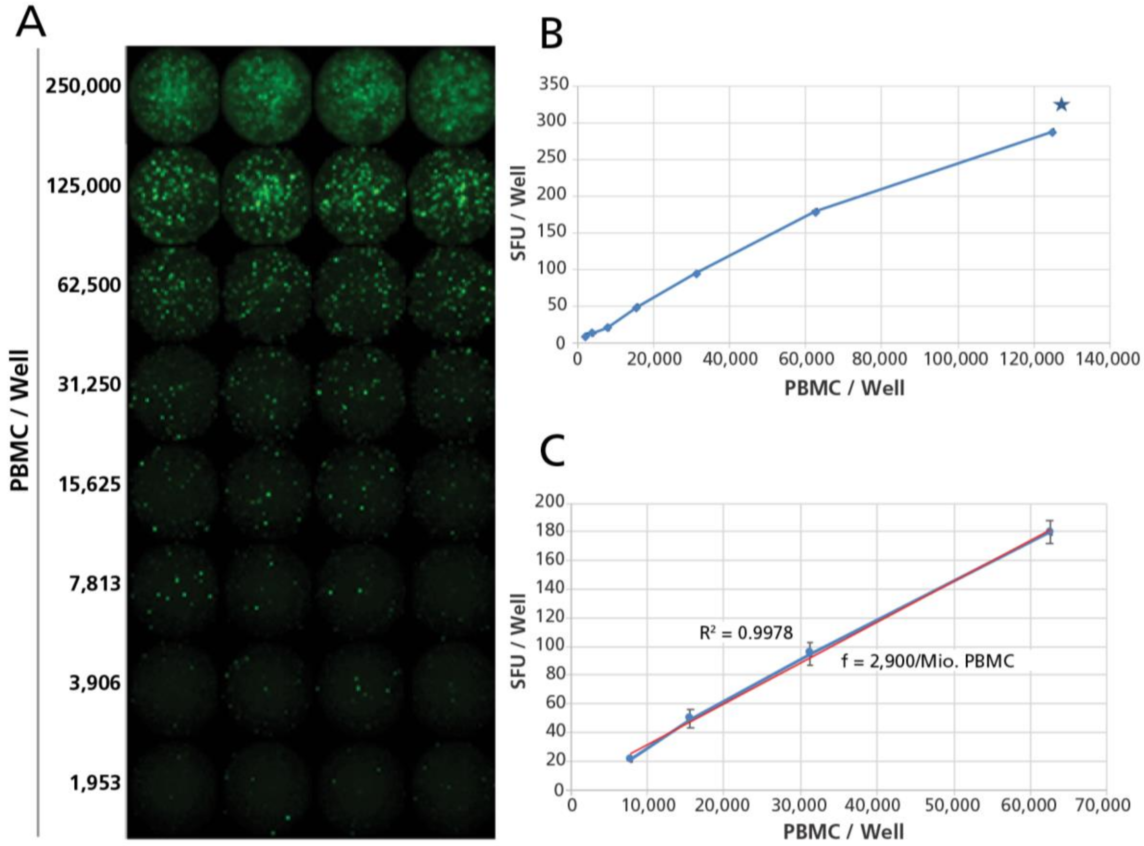

Figure S4. Calculating frequencies for IgA ASC in PBMC illustrated. The legend to Figure 4 applies, except here IgA was detected and analyzed.

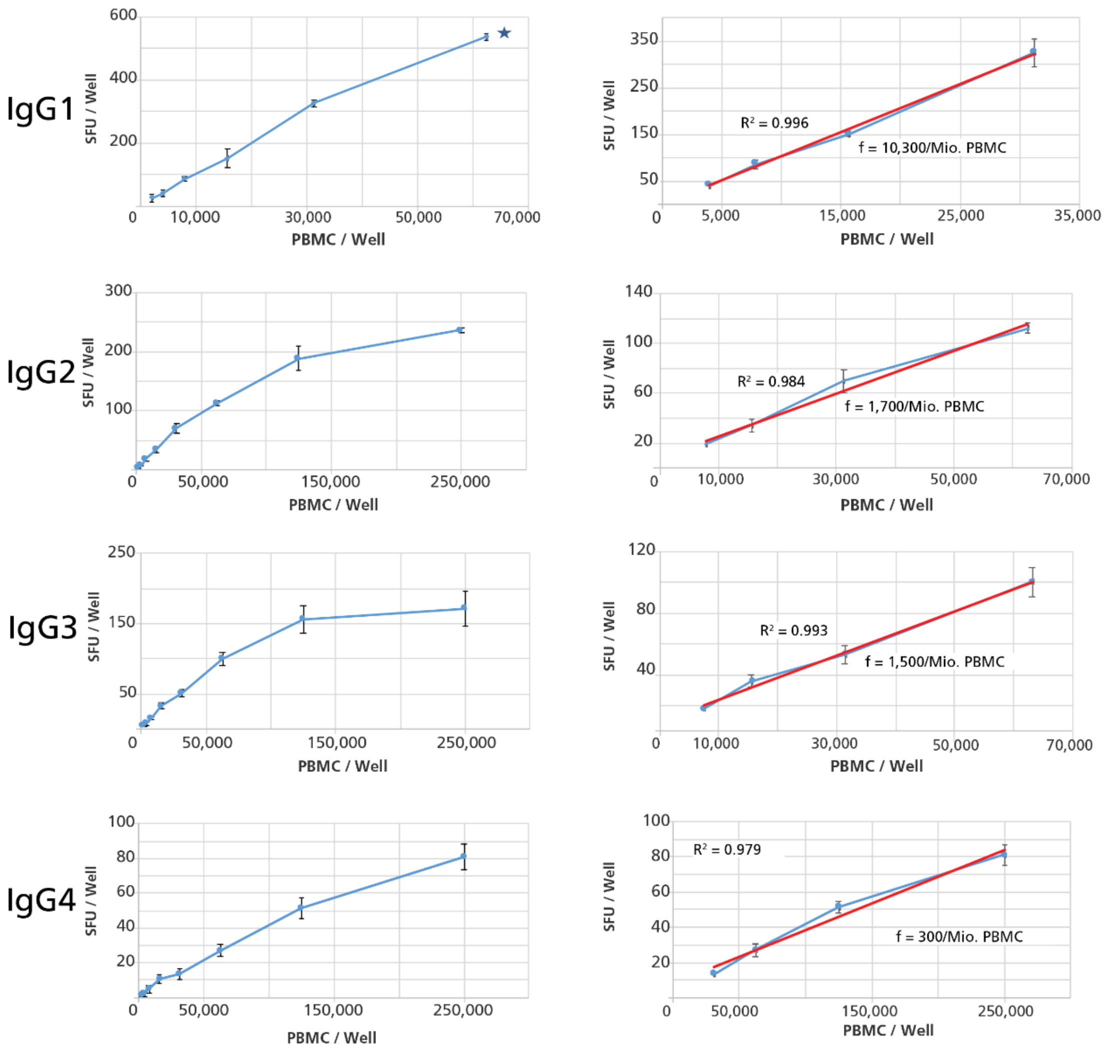

Figure S5. Calculating frequencies for ASC secreting IgG subclasses within PBMC. The legend to Figure 4 applies, except here the four IgG subclasses were detected using a corresponding four color IgG1/IgG2/IgG3/IgG4 ImmunoSpot® assay.
